# Improvement in Serum Magnesium Levels With Sodium-Glucose Cotransporter 2 Inhibitors

**DOI:** 10.1210/jcemcr/luac018

**Published:** 2022-11-30

**Authors:** Arunava Saha, Abdulkadir Omer, Nitin Trivedi

**Affiliations:** PGY2, Internal Medicine, Saint Vincent Hospital, Worcester, MA 01608, USA; Department of Endocrinology and Metabolic Medicine, Saint Vincent Hospital, Worcester, MA, USA; Department of Endocrinology and Metabolic Medicine, Saint Vincent Hospital, Worcester, MA, USA

**Keywords:** hypomagnesemia, SGLT2 inhibitor, magnesiuria, low magnesium, refractory hypomagnesemia

## Abstract

Sodium-glucose cotransporter 2 inhibitors (SGLT2i) are associated with a modest but significant increase in serum magnesium levels. This report describes improvement in serum magnesium and associated symptoms after initiating SGLT2i therapy in a patient with refractory hypomagnesemia. A 58-year-old woman presented with persistent hypomagnesemia refractory to oral magnesium supplements. She had history of type 2 diabetes mellitus, hypothyroidism, fibromyalgia, and degenerative disk disease. The cause of hypomagnesemia was attributed to excessive renal losses. Laboratory investigations revealed serum magnesium of 1.2 mg/dL with fractional excretion of magnesium of 8.9%. She was started on empagliflozin 10 mg daily. Within 4 weeks of therapy, her serum magnesium level corrected with symptomatic improvement, which was sustained a few weeks later. Subsequently, her oral magnesium supplements dose was reduced. SGLT2i has been shown to improve magnesium levels in patients with urinary magnesium wasting. Several mechanisms have been postulated, but the exact physiology remains unknown. SGLT2i have been efficacious for glycemic control, renal protection, decreasing the risk of atherosclerotic cardiovascular disease events, and cardiac mortality in patients with diabetes. In addition, renal and cardiac benefits are also demonstrated in patients without diabetes. This observation demonstrates that SGLT2i can improve the management of patients with otherwise intractable hypomagnesemia.

Hypomagnesemia manifests with a wide variety of abnormalities, including neuromuscular excitability, apathy, cardiac arrhythmias, hypocalcemia, and hypokalemia (1). Additionally, in patients with type 2 diabetes, low magnesium level has been implicated in insulin resistance, pancreatic beta-cell failure, and a higher risk of chronic complications, especially atherosclerotic cardiovascular disease (2). Two major mechanisms that lead to hypomagnesemia include excessive gastrointestinal and renal loss of magnesium. Treating hypomagnesemia is often challenging because of limited gastrointestinal absorption of oral supplements and no reliable agents available to reduce renal losses. Sodium-glucose cotransporter 2 (SGLT2) inhibitors have been associated with a modest but significant increase in serum magnesium levels (3). We present a case of refractory hypomagnesemia from excessive renal loss, which improved with empagliflozin therapy.

## Case Presentation

A 58-year-old woman was referred to endocrinology with persistent symptomatic hypomagnesemia refractory to oral magnesium supplements. She had a history of type 2 diabetes mellitus, class II obesity, hypothyroidism, fibromyalgia, and degenerative disk disease treated with levothyroxine, dulaglutide, and pioglitazone. For the past few years, she had been experiencing symptoms of generalized weakness, malaise, diffuse myalgia, nausea, chronic fatigue, lack of concentration, intermittent holocranial headaches, and occasional dizziness, resulting in significant limitation in her daily activities. She denied drinking alcohol, smoking cigarettes, or using illicit drugs and did not have any diarrhea or weight loss. She also denied use of any over-the-counter medications or proton-pump inhibitors but reported transient improvement in her symptoms with magnesium replacement. Extensive evaluation by nephrology and gastroenterology yielded no clear etiology; the cause was attributed to excessive renal losses, possibly due to familial renal magnesium wasting. She was continued on oral magnesium supplementation with intermittent requirement of intravenous infusions but continued to demonstrate persistent hypomagnesemia with otherwise grossly normal labs. Her physical examination was unremarkable except for symmetrically decreased sensation to fine touch, position sense, and vibration on her lower extremities. Laboratory investigations revealed serum magnesium of 1.2 mg/dL (1.6-2.5 mg/dL). The fractional excretion of magnesium was 8.9% (values >3-4% in the setting of normal renal function and hypomagnesemia indicates excessive renal Mg loss), and glycated haemoglobin of 7.9%. Serum calcium, creatinine, potassium, parathyroid hormone, and thyroid-stimulating hormone were in the normal range (Table 1).

**Table 1.** Investigations for hypomagnesemia and dose of oral magnesium supplements over time

	12/2019	05/2020	11/2020	05/2021	11/2021	03/2022	05/2022	07/2022	08/2022
Magnesium (mg/dL)	1.2	1.1	1.2	1.5	1.5	1.5	1.5	2.2	1.9
Sodium (mg/dL)	140	140	141	136	137	—	143	—	—
Potassium (mg/dL)	4.5	4.1	5.2	4.0	4.0	—	4.8	—	—
Calcium (mg/dL)	10.3	9.4	9.1	9.7	10.0	—	9.9	—	—
Chloride (mg/dL)	102	106	107	101	103	—	105	—	—
Bicarbonate (mg/dL)	22	25	22	23	25	—	21	—	—
Creatinine (mg/dL)	0.92	1.16	0.76	1.01	1.13	—	0.91	—	—
Vitamin D (ng/mL)	65	—	—	—	—	—	—	—	—
Phosphorus (mg/dL)	3.6	—	—	—	—	3.4	—	—	—
Ionized calcium (mg/dL)	5.3	—	—	—	—	—	—	—	—
ESR (mm/hr)	2	—	—	—	—	—	—	—	—
Vitamin B12 (pg/mL)	649	—	—	—	—	—	842	—	—
Folate (ng/mL)	20	—	—	—	—	—	20	—	—
TSH (IU/mL)	1.2	—	3.04	—	3.9	—	1.76	—	—
HbA1c (%)	7.9	7.4	7.1	7.2	6.7	7.1	—	7.0	—
Oral magnesium supplement	Magnesium oxide 400 mg OD	Magnesium chloride 143 mg TID	Magnesium chloride 143 mg TID	Magnesium chloride 143 mg TID	Magnesium chloride 143 mg BID	Magnesium chloride 143 mg BID	Magnesium chloride 143 mg BID	Magnesium chloride 143 mg BID	Magnesium chloride 71.5 mg BID

Abbreviations: BID, twice a day; ESR, erythrocyte sedimentation rate; HbA1C, glycated haemoglobin; TID, 3 times a day; TSH, thyroid-stimulating hormone.

## Treatment

Considering her poor therapeutic response to oral magnesium supplementation, the patient was started on empagliflozin 10 mg daily.

## Outcome and Follow-Up

**Figure 1.**
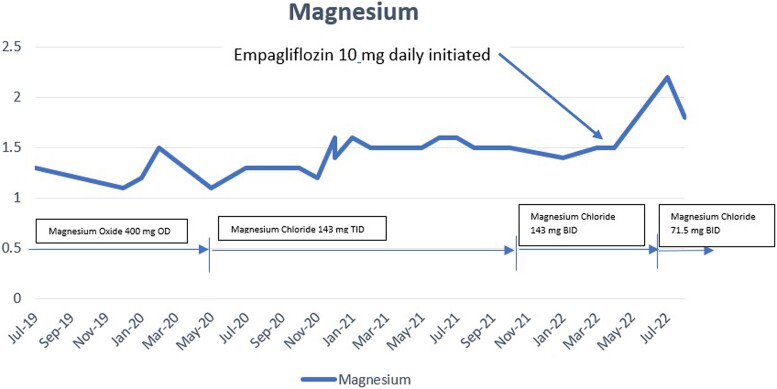
Trend of serum magnesium levels over the years.

Within 4 weeks of therapy, the patient’s serum magnesium level improved to 2.2 mg/dL, with sustained improvement demonstrated on repeat laboratory evaluation a few weeks later (Fig. 1). She also reported reduced fatigue and myalgia accompanied by improved concentration and mental focus. Her oral magnesium supplements dose was reduced to half, which she tolerated well.

## Discussion

Magnesiuria in patients with diabetes mellitus results from the reduced activity of the transient receptor potential melastatin 6 (TRRPM6) ion channel in the distal convoluted tubules, perhaps related to a combination of factors including insulin resistance, glycosuria, and altered glucagon secretion (2). Long-term treatment of hypomagnesemia with oral magnesium supplementation is usually unsuccessful because of limited gastrointestinal absorption and the risk of diarrhea with higher supplemental doses of magnesium (4). Unfortunately, there are no good therapeutic options for decreasing renal loss of magnesium. Potassium-sparing diuretics such as amiloride have been used previously in the management of refractory hypomagnesemia but failed to demonstrate consistent benefits (5).

SGLT2 inhibitors have been shown to improve magnesium levels in patients with urinary magnesium wasting (6). A meta-analysis of patients with type 2 diabetes receiving SGLT2 inhibitors improved hypomagnesemia with a mean increase in serum magnesium levels of 0.01 to 0.24 mg/dL. Reduction in fractional excretion of magnesium has been observed, signifying enhanced tubular reabsorption (3).

Several mechanisms have been postulated for improved renal magnesium handling with SGLT2 inhibition, including decreasing cotransport of sodium and glucose in the proximal tubule causing increased intraluminal electrical potential leading to magnesium reabsorption and extracellular fluid volume depletion induced renin-angiotensin-aldosterone system activation enhancing tubular magnesium uptake through solvent drag. Other possible mechanisms include increased expression of magnesium transporters in the kidney or gut, hypertrophy or hyperplasia of tubular magnesium transporting segments, and altered production of signaling agents such as epidermal growth factor influencing magnesium reabsorption. SGLT2 inhibitor-associated reduction of glomerular filtration due to tubuloglomerular feedback, causing increased afferent arteriolar and reduced efferent arteriolar tone, can also reduce urinary magnesium loss. SGLT2-induced decrease in insulin resistance and increase in glucagon levels may also play a role in increasing transient receptor potential melastatin 6-mediated distal tubular magnesium resorption (7). Whether these effects persist over time remains unclear.

SGLT2 inhibitors in patients with diabetes mellitus have been efficacious for glycemic control, renal protection, decreasing the risk of atherosclerotic cardiovascular disease events, and reducing cardiac mortality (8). In addition, SGLT2 inhibitors have been shown to improve renal and cardiac outcomes in patients without diabetes mellitus (9). Larger efficacy trials are necessary to assess whether SGLT2 inhibitors are consistently effective in hypomagnesemia from different etiology.

## Data Availability

Data sharing is not applicable to this article as no data sets were generated or analyzed during the current study.

## Learning Points

- Hypomagnesemia can have a wide variety of presentations and is often difficult to treat.
- SGLT2 inhibitors have been shown to improve serum magnesium levels in patients with refractory hypomagnesemia with several different mechanisms of action.
- SGLT2 inhibitors are efficacious in diabetes mellitus and also improve renal and cardiac outcomes, but further trials are necessary to assess consistent efficacy in hypomagnesemia.
